# Molecular Diagnosis of Footrot and Contagious Ovine Digital Dermatitis in Small Ruminants in the Iberian Peninsula

**DOI:** 10.3390/ani14030481

**Published:** 2024-02-01

**Authors:** Alfredo A. Benito, Silvia Anía, María de los Ángeles Ramo, Cristina Baselga, Joaquín Quílez, María Teresa Tejedor, Luis Vicente Monteagudo

**Affiliations:** 1 Exopol S.L., Pol Río Gállego D/14, San Mateo de Gállego, 50840 Zaragoza, Spain; abenito@exopol.com (A.A.B.); sania@exopol.com (S.A.); crbaselga@exopol.com (C.B.); 2 Department of Animal Pathology, Faculty of Veterinary Sciences, University of Zaragoza, 50013 Zaragoza, Spain; mrgvet@unizar.es; 3 Agrifood Institute of Aragón (IA2), University of Zaragoza-Centro de Investigación y Tecnología Alimentaria de Aragón (CITA), 50013 Zaragoza, Spain; jquilez@unizar.es; 4 Department of Anatomy, Embriology and Genetics, Faculty of Veterinary Sciences, University of Zaragoza, 50013 Zaragoza, Spain; ttejedor@unizar.es; 5 Aragon Institute of Health Sciences (IACS), Centro de Investigación Biomédica en Red-Enfermedades Cardiovasculares (CIBERCV), 50009 Zaragoza, Spain

**Keywords:** ovine, caprine, contagious ovine digital dermatitis, footrot, qPCR, *Dichelobacter nodosus*, *Fusobacterium necrophorum*, *Treponema phagedenis*, *Treponema medium*, *Treponema pedis*

## Abstract

**Simple Summary:**

Contagious ovine digital dermatitis (CODD) and footrot (FR), a sub-acute or acute necrotic (decaying) infectious disease involving the hoof and underlying tissues, cause important economic losses all over the world. The aetiological agent for FR is *Dichelobacter nodosus* while CODD has been associated with pathogenic *Treponema* phylogroups. In the present work, we applied a set of quantitative polymerase chain reactions to detect in a set of 105 samples from diseased flocks and 15 from apparently healthy herds the following microbiological agents: *Dichelobacter nodosus*, *Fusobacterium necrophorum*, *Treponema* spp., and three pathogenic *Treponema* phylogroups (*T. phagedenis, T. medium*, and *T. pedis*). For *Treponema* spp., all the samples gave a positive result, so we focused our interest on the other agents. *D. nodosus* and *F. necrophorum* are the most frequently found bacteria (alone or in combination). Although *F. necrophorum* is nowadays known to be not the causative agent for FR, it is the only detected agent in 16.2% of the cases, and it is important to carry out analysis to identify it in order to allow veterinarians to apply the correct prevention and treatment procedures. Multiple serogroups of *D. nodosus* are often (35.1%) found in the pools. This report concludes with comprehensive proposals for diagnosing and preventing hoof ailments in ovine and caprine herds.

**Abstract:**

Contagious ovine digital dermatitis (CODD) and footrot (FR), a sub-acute or acute necrotic (decaying) infectious disease involving the hoof and underlying tissues, pose economic challenges to herds in Spain and worldwide. The aetiological agent for FR is *Dichelobacter nodosus*, while CODD is caused by pathogenic *Treponema* phylogroups. We detail the findings derived from the analysis by qPCR of 105 pooled samples from 100 ovine and five caprine herds in Spain and Portugal, alongside 15 samples from healthy flocks in order to identify *Dichelobacter nodosus*, *Fusobacterium necrophorum*, *Treponema* spp., and three pathogenic *Treponema* phylogroups (*T. phagedenis*, *T. medium*, and *T. pedis*). *Treponema* spp. were detected in all 120 pools, including samples from the 15 healthy flocks where only one positive result for *F. necrophorum* was recorded. Mixed infections by agents different from *Treponema* spp. were identified in 68.57% of samples. Positive results for *F. necrophorum* and/or *D. nodosus*, were obtained for 91.4% of the pools, whereas the presence of the three pathogenic *Treponema* phylogroups was rare: each of them appeared in isolation in a single pool, while they were found in 18 pools in combination with other agents. While *F. necrophorum* was the sole finding in 16.2% of samples from affected herds, *D. nodosus* (the footrot causative agent) was only detected in 61% of affected farms. An improved qPCR protocol was implemented to determine the serogroups of *D. nodosus* in the samples and found all of them (except the G serogroup), often in combined infections (35.1%). This report concludes with comprehensive proposals for diagnosing, preventing, and treating hoof ailments, remarking the interest of the information about *D. nodosus* serogroups in order to improve the efficiency of immunization by choosing appropriate vaccine protocols.

## 1. Introduction

The etiology of ovine infectious foot diseases is still under active investigation. Generally, these diseases are attributed to two main pathologies: contagious ovine digital dermatitis (CODD), and footrot (FR). Traditionally, digital dermatitis (DD) has been associated with pathogenic *Treponema* infections in both cattle and sheep, and the same agents have been confirmed to be in the origin of severe DD lesions in goats [1,2]; on the other hand, footrot is a highly contagious hoof ailment affecting both sheep and goats. It is primarily caused by virulent strains of *Dichelobacter nodosus*, a gram-negative bacterium that thrives in anaerobic conditions. Currently, 10 distinct serogroups of *D. nodosus* are recognized based on fimbrial antigens (A–I and M), and it is important to note that there is no cross-immunity between them. While *Fusobacterium necrophorum* was previously believed to play a primary role in this disease, recent research has clarified its role as an opportunistic bacterium that exacerbates the severity and duration of hoof lesions [3,4,5].

However, some researchers suggest the coexistence of the agents responsible for FR and *Treponema* sp. in different healthy and diseased conditions [6,7].

The pathogenesis and timing of bacterial colonization of the hoof and other foot areas are still under investigation, even though most cases are assumed to have a poly-bacterial origin, including the above mentioned *D. nodosus*, *F. necrophorum*, and three digital dermatitis *Treponema* phylogroups (*T. phagedenis*, *T. medium*, and *T. pedis*). Additionally, various environmental factors, such as moisture, temperature, and previous injuries, play critical roles in the onset of these diseases [7,8,9,10].

Despite the continuous reduction in the sheep population in Spain (48.5% and 65% between 2007 and 2023 for meat sheep and dairy sheep, respectively), and a decline in caprine numbers (10.4% between 2007 and 2023), the sector still represents a significant economic activity [11,12].

For decades, hoof ailments in sheep and goats have been major causes of economic losses in Spain and worldwide. In 1987, losses due to FR in Spanish sheep farming were estimated at 1765 million pesetas (equivalent to 10.61 million euros at the exchange rate of 166.386 pesetas/euro) [13]; in fact, FR is a global problem, with disease costs reaching 24 million pounds in the United Kingdom, 18.4 million dollars in Australia and 11 million dollars in New Zealand [14,15,16].

In this study, the bacterial etiology of FR and CODD was investigated using qPCR assays targeting the main pathogens associated with podal affections in domestic small ruminants, including *D. nodosus* and its specific serogroups, *F. necrophorum and Treponema* sp., and three pathogenic phylogroups: *T. pedis*, *T. phagedenis*, and *T. medium*. For this purpose, pooled samples from both clinical cases (n: 105) and from healthy herds (n: 15) from Spain and Portugal, submitted between 2019 and 2022 to a veterinary diagnostic laboratory, were analyzed. Additionally, recommendations for the diagnosis of foot disease are provided.

## 2. Materials and Methods

### 2.1. Ethical Concerns

Animal care and use committee approval were not necessary for this study, as it falls under the category of non-experimental clinical veterinary practices, conducted with the agreement of animal owners and for diagnostic purposes.

### 2.2. Swab Samples

Foot swabs from sheep and goat farms affected by foot ailments in Spain (n: 93) and Portugal (n: 12) were submitted for microbiological diagnostic purposes to a specialized veterinary laboratory (Exopol S.L., San Mateo de Gállego, Spain). Different veterinary practitioners submitted a total of 105 pooled samples: each pool proceeded from a single herd and consisted of five swabs and each of them were obtained from one affected hoof of a single individual so that the number of pools was coincident with the number of herds (containing 100 ovine and five caprine flocks). Indicatively, it was recommended to sample animals without advanced or chronic lesions, preferably similar to those corresponding to score 2–3 to avoid bacterial contamination, using the guide freely available at https://footrotsydney.org/foot-scores/ (last accession 18 December 2023) [17]. However, neither detailed information on the lesions observed in each individual nor a previous clinical diagnostic for the appearing disease were submitted to the laboratory by the veterinarians.

Age information was available for 89 pools, predominantly from adult animals, so the statistical analysis of this parameter is not suitable. Also, the information about the production performed in each farm of origin is only partially available: a total of 18 farms are known to be devoted to meat production while 14 are confirmed to be involved in milk production.

Additionally, 15 pooled samples from 15 different unaffected farms (five swabs per pool, each swab obtained from a single hoof of a single healthy adult individual) were obtained and used to evaluate the presence of these agents in these types of animals (control group).

### 2.3. Pathogen Identification

The pooled samples were subjected to the extraction of nucleic acids using the commercial kit MagMAX™ Pathogen RNA/DNA (Thermo Fisher Scientific, Waltham, MA, USA) with an automated magnetic particle processor (KingFisher Flex; Thermo Fisher Scientific) according to the manufacturer’s instructions.

The molecular detection of *F. necrophorum*, *D. nodosus*, virulent/avirulent strains of *D. nodosus*, and 10 *D. nodosus* serogroups (A to I and M) were achieved using commercial qPCR kits (EXOone qPCR, Exopol S.L., Spain) following the manufacturer’s instructions available at https://www.exopol.com/es/exoone/manuals.php (last accession 14 January 2024). An endogenous control was also included in these assays to avoid false-negative results, and the sample was considered positive when the quantification cycle (Cq) was <38 according to the user´s manual of these kits. The identification of *Treponema* spp. and pathogenic phylogroups (*T. pedis*, *T. medium*, and *T. phagedenis*) were performed using the previously described qPCR assays, based upon specific PCR primers and fluorescent probes [18]. The target gene of each qPCR assay and its coded proteins are shown in Table 1. 

### 2.4. Statistical Procedures

Statistical analyses were performed using IBM SPSS Statistics 26.0 software. The Pearson chi square test and, alternatively, the Fisher exact test were used for ascertaining the association between agents (presence/absence) and status (clinical case/control). The phi coefficient was also calculated as a measure of the association between these two binary variables. For each agent, the comparison of Cq values between simple and combined detections was carried out by a one-way analysis of variance (ANOVA). *p*-values < 0.050 were considered statistically significant.

## 3. Results

### 3.1. Identification of Bacteria Species in the Samples

All samples from both healthy and affected animals yielded positive results for *Treponema* spp. Given the high positivity for *Treponema* spp. in our dataset, only the samples testing positive for pathogenic *Treponema* phylogroups, including *Treponema pedis*, *Treponema medium*, and *Treponema phagedenis* (known causative agents of bovine digital dermatitis and CODD), are included in the following analysis of single and multiple infections.

Other than *Treponema* spp., only *F. necrophorum* was obtained in one of the 15 pools from the control group.

All but six samples from the diseased herds tested positive for any of the pathogenic bacterial species examined in this study, including *F. necrophorum*, *D. nodosus*, and the three pathogenic *Treponema* phylogroups (Table 2). Due to the small number of caprine samples, they were not analyzed in a separate way: in fact, one of these five caprine samples provided negative results for all the tests (except for *Treponema* spp.) while the rest were positive for different pathogens combinations.

The presence of a single pathogen was reported in a quarter of these samples, with *F. necrophorum* being the most common (16.2%).

A polybacterial pathogen etiology was found in 68.57% of these samples; it is significant that the combination of only *F. necrophorum* and *D. nodosus* was found in more than half of the samples and combinations including at least one of these two bacteria, and one or more pathogenic *Treponema* phylogroups were found in 17.1% of the specimens. Most submissions (96/105, 91.4%) yielded positive results for *F. necrophorum* and/or *D. nodosus*, with the sporadic presence of one or more of the three pathogenic *Treponema* phylogroups. Overall, *F. necrophorum* was identified in 89 (84.8%) samples, and 64 (60.9%) samples tested positive for *D. nodosus*, with pathogenic *Treponema* being reported in 21 (20%) samples.

Even if *D*. *nodosus* is the causative agent of FR, at the time of sampling only 61.0% (64/105) of the clinical cases were positive for *D*. *nodosus*, while no control showed this agent (*p* < 0.001); an intermediate association for foot ailments and *D*. *nodosus* is therefore ascertained (phi coefficient = 0.404; *p* < 0.001). On the other hand, *F. necrophorum* was present in 84.8% (89/105) of clinical cases and in 6.7% (1/15) of the controls (*p* < 0.001); the association between foot ailments and this agent was more intense (phi coefficient = 0.596; *p* < 0.001). 

It is important to note that no statistically significant differences were observed in the values of Cq obtained for each agent in single and combined detections. Also, the Cq values for *Treponema* spp. in both healthy and affected individuals were not significantly different.

### 3.2. D. nodosus Serogroups

In our study, we successfully identified *D. nodosus* serogroups in 57 samples that had tested positive for this agent. This information was specifically requested by the submitting veterinarians, and the detailed results can be found in Table 3. The serogroups B and D were the most frequent ones with 35% (20/57) and 33% (19/57) of positive cases, respectively. Monovalent infection by serogroups A, B and C accounts for 36% of the total serotyped cases.

## 4. Discussion

The 100% positivity for *Treponema* spp. closely mirrors recent findings in Sweden, where 90.6% of feet from slaughtered lambs in the autumn of 2021 tested positive in this test, too, even if only 10 out of 512 lambs exhibited lesions compatible with FR or CODD [19]. It is important to note that most animals sampled in the current study were adults, while only slaughter lambs were analyzed in the Swedish work. This finding also aligns with an earlier study on the environments of bovine farms affected by bovine digital dermatitis (BDD). In that investigation, a *Treponema-*genus-specific assay produced affirmative outcomes in 92.9% of the slurry samples, all fecal samples, and 80% of urine samples, despite the absence of BDD-specific pathogenic *Treponema* in these sample types [20]. In all, these results point to the limited (if any) practical utility of the detection of *Treponema* spp. (different from pathogenic phylogroups) for veterinary activity in the field. 

The 16.2% of positive samples in the present set of data for only *F. necrophorum* highlights the potential role of this bacterium as an agent that contributes to the severity and duration of footrot in sheep and goats. This agent is present in 84.8% of the samples submitted from diseased farms. Two previous studies performed in Spain found *F. necrophorum* in 5% and in 8.8% of the isolated strains from footrot samples [21,22]. This difference in the proportions could be due to the different methodology: the present work is not based on isolated strains but on nucleic acids detected directly from the pooled samples. The interest in maintaining *F. necrophorum* detection protocol is evident, even if (as mentioned above) the origin of FR depends on *D. nodosus*. Both bacteria are associated with sheep. However, *D. nodosus* persists only in the soil and in diseased animals, not in healthy feet, while *F. necrophorum* persists in footrot-diseased feet and in mouth and feces (different strains in mouths and feces). For this reason, the elimination of *F. necrophorum* is a more challenging task [23]. 

The results of the current study support the coexistence of *D*. *nodosus*, *F. necrophorum*, and pathogenic *Treponema* phylogroups based on observations of ovine foot dermatitis and necrosis in the field: this could be extremely relevant for the practitioners submitting samples to the diagnostic laboratories in order to establish efficient treatment and prophylaxis procedures. Moreover, multiple studies have highlighted the intricate interactions among various agents in ovine foot disease. In a study of two CODD outbreaks in Sweden, the coexistence of *Treponema* spp., *F. necrophorum*, and *D. nodosus* was confirmed by molecular tests, even if the presence of BDD-associated *Treponema* phylogroups could not be confirmed by molecular procedures [24]. In a recent experiment, sheep were inoculated with tissue materials obtained from the hoof lesions of elks affected by digital dermatitis. Positive PCR results were obtained not only for pathogenic *Treponema* phylogroups but also for *D*. *nodosus* in two inoculated individuals, while *F. necrophorum* was detected in animals from both the inoculated and mock groups [25]. The simultaneous presence of these three genera of agents was also confirmed in a recent independent study, based on 254 ovine foot lesions and 15 apparently healthy ovine feet, suggesting that this combination plays a substantial role in influencing the severity of the lesions. Even if the authors categorize the cases showing pathogenic *Treponema* (alone or combined with other agents) as CODD, the co-existence with *D. nodosus* and *F. necrophorum* is evidenced in 27.4% and 54.4% of them, respectively. On the other hand, in pathogenic *Treponema* negative samples, *D. nodosus* and *F. necrophorum* appear in 11.1% and 14.12% of the cases, respectively. The authors conclude that these agents are significantly associated and their different combinations influence the severity of the lesions [26].

Recent research has emphasized a dynamic and complex microbiome approach in the study of foot pathology in bovines. The results of a comprehensive study on interdigital microbiota conducted over a span of 20 weeks suggest that dysbiosis plays a pivotal role in the initial stages, where seven operational taxonomic units (OTUs) may serve as valuable predictors for evaluating a sheep’s predisposition to *D*. *nodosus* colonization and, subsequently, to the onset of FR. The microbiota undergoes dynamic changes, and significant alterations also occur during the incubation period as the feet progress towards disease manifestation [8].

Even though our sample size is limited, the data analysis provides valuable insights into the microbiological complexity of *D. nodosus* infections. Notably, all 10 serogroups were found, except for serogroup G. Among the 57 determinations carried out, a single serogroup was identified in 37 cases (64.9%), while the remaining 20 samples (35.1%) exhibited various combinations of up to four serogroups. 

Our results, obtained after nucleic acid extraction from pools of swabs, confirm that infections by a single *D. nodosus* serogroup are not a constant occurrence, as has been previously reported by different groups [27,28,29]. 

In the present study, serogroups B and D of *D. nodosus*, either alone or in combination with other serogroups, were the most frequently found (20/57, 35.1% and 19/57, 33.3%, respectively), followed by serogroup C (12/57, 19.3%), E (9/57, 15.8%) and A (8/57, 14%). Lower frequencies were obtained for serogroups F (5/57, 8.7%), H (6/57, 10.5%) and I (1/57, 1.7%). Moreover, serogroup M was observed, either alone or in combination, in three samples submitted from Portugal (3/57, 5.26%). These results are not totally concordant with those of two previous publications in our country. The first one indicated that serogroups A and C were predominant, while serogroup D accounted for only 1.8% of the cases [22]. A more recent serological study in the Spanish region of Extremadura yielded similar results: serogroups A and C were identified in 40.7% and 25.9% of the cases, respectively [30]. Both reports were based on the previous isolation of bacterial strains, followed by immunological microagglutination with specific sera. As it can be seen, a wide time lapse and very different methodological approaches could explain these different results. To the authors’ knowledge, our study is the first report of the molecular serogrouping of *D. nodosus* on clinical cases in Spain. As far as we know, this is the first report of the detection of the *D. nodosus* M serogroup in Portugal. A previous study performed by another group in that country did not detect this serogroup [28]. 

A survey in England found serogroups H and B present separately in 60% of the studied flocks and in combination in 27% of the farms. Nevertheless, in the same report, a total of 50 combinations of different serogroups (A–I) were observed in the 164 flocks involved in the study [31]. In Germany, a study involving 969 isolates from 83 farms identified the serogroup A as the most prevalent (38.1%), followed by B (31.1%) and H (21.4%), with G (not found in our study) accounting for 16.1% [28]. In contrast, a survey conducted in the Jammu and Kashmir territory of India did not find serogroup A, while 78.7% of the samples were assigned to serogroup B [32]. It is important to state that the four previously cited studies from Portugal, England, Germany, and India were based on the serogrouping of isolated strains using the widely applied, conventional PCR procedure described in 2002 by Dhungyel et al. [33]. In the present study, the qPCR procedure used updates the molecular characterization of *D. nodosus* by applying specific molecular probes to identify the existing fimbrial variants. This new tool allows direct serogrouping on clinical samples, thereby avoiding the possible bias associated with the choice of isolated colonies.

Also, these variations could be attributed to geographical distances or to differences in environmental conditions, such as moisture and temperature, although no difference in virulence has been observed among the different *D. nodosus* serogroups [34].

The presence of several serogroups of *D. nodosus* in an affected flock is relevant in order to apply appropriate prevention measures. The existence of antigenic competition among the different serogroups limits the efficacy of multivalent vaccines [35]. On the other hand, when the efficiency of multivalent vaccines is compared to that of serial inoculations of bivalent vaccines, it is concluded that a two-month interval is sufficient to avoid antigenic competition and to achieve effective immunization [27]. In our study, around 65% of clinical cases showed infection by only one serogroup (Table 3); considering the described antigenic competition, a significant number of farms in our study could improve their current strategies of immunization using monovalent or bivalent vaccines.

In the present work, we have used commercial qPCR assays, which were validated to detect the 10 existing serogroups, including M. In a recent study, a qPCR was also designed in order to detect all of the serogroups but, in the absence of M samples in their study, the authors have not yet verified its ability to detect this serogroup [36].

Several reports have emphasized the importance of identifying serogroups to establish effective vaccination protocols [31,32,34]. With reference to the M serogroup, it has been reported to have been successfully used in vaccination protocols in at least one case [37]. 

Veterinary practice in the field is inherently complex, as numerous factors are beyond control. Veterinarians are often called by owners when lameness and/or necrosis affect a significant portion of a flock. At this point, it is impossible to determine the exact timing of an infection, the evolution of the lesions, and the potential changes in microbiota.

The laboratory analysis yielded totally negative microbiological results for six of the swab pools, even if such pools had always been obtained from diseased feet. There are several possible explanations for this situation. First, the observed symptoms or lesions could be unrelated to either CODD or FR. For instance, in laminitis, white line disease, injuries, or ergotism, bacterial infections are not supposed to be the origins of the feet diseases [38]. On the other hand, it seems difficult to accept that the incorrect sampling and transport of the samples is the origin of the negative results, even if it has been demonstrated that the swabs may contain only a fraction of the bacteria existing in a simulated exudate [39]. 

## 5. Conclusions

As it refers to the precise diagnosis and denomination of the hoof ailment in a particular herd, the focus should be on qPCR identification of the disease-causing agents to prescribe appropriate treatment and profilaxis. Our results point to a cost-effective procedure for identifying the infectious agents involved in feet ailments in herds localized at a significant geographical distance of the laboratories: pooling five swabs from five different animals and testing with qPCR for *D*. *nodosus*, *F. necrophorum*, and the three pathogenic *Treponema* phylogroups. In the absence of precise, detailed information on this subject, one should test for these agents. On the other hand, the utility of *Treponema* spp. PCR in adult individuals is very limited (if there is any) because all the samples in our study (both affected and controls) provided positive results for this test. Considering the previous reports about the existence of an antigenic competition among serogroups, the characterization of *D. nodosus* serogroups involved in each outbreak could be a useful tool for establishing precise vaccine programs: in the light of our results, a significant proportion of the herds could improve their immunization procedures by choosing the appropriate monovalent or bivalent vaccines. 

## Figures and Tables

**Table 1:** Etiological agent, target gene, and coded protein identified by the qPCR kit used. For the commercial kits, the information on validation tests is available at https://www.exopol.com/es/exoone/manuals.php (last accession 19 December 2023).

Etiologial Agent	Target Gene	Coded Protein	Reference
*Fusobacterium necrophorum*	lktA	Leukotoxin A	Commercial kit
*Dichelobacter nodosus*	16S	D16S rRNA	Commercial kit
*D. nodosus virulent strains*	aprV2	Acidic protease 2	Commercial kit
*D. nodosus non virulent strains*	aprB2	Acidic protease 2	Commercial kit
*D. nodosus serogroups*	fimA	Fimbrial protein	Commercial kit *
*Treponema* spp.	16S	D16S rRNA	Anklam et al. 2017 [18]
*Treponema pedis*	16S-tRNA region	16S rRNA intergenic space	Anklam et al. 2017 [18]
*Treponema phagenedenis*	16S-tRNA region	16S rRNA intergenic space	Anklam et al. 2017 [18]
*Treponema medium*	flaB2	Flagellar filament 31.3 kDa	Anklam et al. 2017 [18]

* Ten different commercial qPCR kits for serogroups: A, B, C, D, E, F, G, H, I, and M.

**Table 2:** Bacterial species identified in foot swabs from 105 clinically affected ovine and caprine herds submitted to a veterinary diagnostic laboratory in Spain [number positive (%)].

Pathogen Agent	Count/n	%
None	6/105	5.71
*D. nodosus*	7/105	6.67
*F. necrophorum*	17/105	16.19
*T. pedis*	1/105	0.95
*T. medium*	1/105	0.95
*T. phagedenis*	1/105	0.95
*D. nodosus + F. necrophorum*	54/105	51.45
*D. nodosus + F. necrophorum + T. pedis*	1/105	0.95
*D. nodosus+ F necrophorum+ T. pedis + T. phagedenis*	2/105	1.90
*F. necrophorum + T. pedis*	4/105	3.81
*F. necrophorum + T. pedis + T. medium*	4/105	3.81
*F. necrophorum+ T. pedis + T. phagedenis*	2/105	1.90
*F. necrophorum+ T. pedis +T. medium + T. phagedenis*	5/105	4.76

**Table 3:** Distribution of the *D. nodosus* serogroups detected in the present study [number positive (%)].

Serotype	Count/n	%
A	(4/57)	7.02
B	(9/57)	15.8
C	(7/57)	12.3
D	(6/57)	10.5
E	(4/57)	7.02
F	(3/57)	5.26
H	(1/57)	1.75
I	(1/57)	1.75
M	(2/57)	3.51
A + B + E + H	(1/57)	1.75
A + C	(1/57)	1.75
A + C + D	(1/57)	1.75
A + D	(1/57)	1.75
B + C + D	(1/57)	1.75
B + D	(3/57)	5.26
B + D + M	(1/57)	1.75
B + E	(1/57)	1.75
B + E + H	(1/57)	1.75
B + H	(3/57)	5.26
C + D	(2/57)	3.51
D + E	(2/57)	3.51
D + F	(2/57)	3.51
**Count/n**	57/57	

## Data Availability

The data presented in this study are available on request from the corresponding author. The data are not publicly available due to the Privacy Policy of the Company.

## References

[1] Crosby-Durrani H.E., Clegg S.R., Singer E., Angell J.W., Evans N.J., Carter S.D., Blundell R.J., Duncan J.S. (2016). Severe Foot Le-sions in Dairy Goats Associated with Digital Dermatitis Treponemes. J. Comp. Path..

[2] Sullivan L.E., Evans N.J., Clegg S.R., Carter S.D., Horsfield J.E., Duncan J.S., White D.G. (2015). Digital dermatitis treponemes associated with a severe foot disease in dairy goats. Veter. Rec..

[3] Egerton J.R., Roberts D.S., Parsonson I.M. (1969). The aetiology and pathogenesis of ovine foot-rot. I. A histological study of the bacterial invasion. J. Comp. Pathol..

[4] Roberts D.S., Egerton J.R. (1969). The aetiology and pathogenesis of ovine foot-rot. II. The pathogenic association of *Fusiformis nodosus* and *Fusiformis necrophorus*. J. Comp. Pathol..

[5] Zanolari P., Dürr S., Jores J., Steiner A., Kuhnert P. (2021). Ovine footrot: A review of current knowledge. Veter. J..

[6] Duncan J.S., Angell J.W., Richards P., Lenzi L., Staton G.J., Grove-White D., Clegg S., Oikonomou G., Carter S.D., Evans N.J. (2021). The dysbiosis of ovine foot microbiome during the development and treatment of contagious ovine digital dermatitis. Anim. Microbiome.

[7] Maboni G., Frosth S., Aspán A., Tötemeyer S. (2016). Ovine footrot: New insights into bacterial colonization. Vet. Rec..

[8] Clifton R., Monaghan E.M., Green M.J., Purdy K.J., Green L.E. (2022). Differences in composition of interdigital skin microbiota predict sheep and feet that develop footrot. Sci. Rep..

[9] Angell J., Grove-White D., Duncan J. (2015). Sheep and farm level factors associated with contagious ovine digital dermatitis: A longitudinal repeated cross-sectional study of sheep on six farms. Prev. Veter. Med..

[10] Staton G.J., Angell J.W., Grove-White D., Clegg S.R., Carter S.D., Evans N.J., Duncan J.S. (2021). Contagious Ovine Digital Dermatitis: A Novel Bacterial Etiology and Lesion Pathogenesis. Front. Veter. Sci..

[11] Ministerio de Agricultura, Pesca y Alimentación Indicadores Económicos del Sector Ovino y Caprino de Carne. https://www.mapa.gob.es/es/ganaderia/temas/produccion-y-mercados-ganaderos/indicadoreseconomicosdelsectorovinoycaprino_carne_2023a_tcm30-511496.pdf.

[12] Ministerio de Agricultura, Pesca y Alimentación Ovino de Leche, Junio de 2023. https://www.mapa.gob.es/es/ganaderia/temas/produccion-y-mercados-ganaderos/dashboardovinoleche_junio2023_tcm30-428244.pdf.

[13] Berga A.M., Sanchez P. (1990). Incidencia económica de la Sanidad en ovino. Programas sanitarios básicos. Mundo Ganadero.

[14] Green L.E., George T.R.N. (2008). Assessment of current knowledge of footrot in sheep with particular reference to Dichelobacter nodosus and implications for elimination or control strategies for sheep in Great Britain. Vet. J..

[15] Raadsma H., Dhungyel O. (2013). A review of footrot in sheep: New approaches for control of virulent footrot. Livest. Sci..

[16] Angell J.W., Duncan J.S., Carter S.D., Grove-White D.H. (2014). Farmer reported prevalence and factors associated with contagious ovine digital dermatitis in Wales: A questionnaire of 511 sheep farmers. Prev. Vet. Med..

[17] Egerton J., Roberts D.S. (1971). Vaccination against ovine foot-rot. J. Comp. Pathol..

[18] Anklam K., Kulow M., Yamazaki W., Döpfer D. (2017). Development of real-time PCR and loop-mediated isothermal amplification (LAMP) assays for the differential detection of digital dermatitis associated treponemes. PLoS ONE.

[19] Rosander A., Albinsson R., König U., Nyman A., Frosth S. (2022). Prevalence of bacterial species associated with ovine footrot and contagious ovine digital dermatitis in Swedish slaughter lambs. Acta Veter. Scand..

[20] Evans N.J., Timofte D., Isherwood D.R., Brown J.M., Williams J.M., Sherlock K., Lehane M.J., Murray R.D., Birtles R.J., Hart C.A. (2012). Host and environmental reservoirs of infection for bovine digital dermatitis treponemes. Veter. Microbiol..

[21] Piriz S., Cuenca R., Valle J., Vadillo S. (1990). Isolation and identification of anaerobic bacteria from ovine foot rot in Spain. Res. Vet. Sci..

[22] Hurtado M.A., Píriz S., Valle J., Jimenez R., Vadillo S. (1998). Aetiology of ovine footrot in Spain. Veter. Rec..

[23] Clifton R., Giebel K., Liu N.L.B.H., Purdy K.J., Green L.E. (2019). Sites of persistence of *Fusobacterium necrophorum* and *Dichelobacter nodosus*: A paradigm shift in understanding the epidemiology of footrot in sheep. Sci. Rep..

[24] Bernhard M., Frosth S., König U. (2021). First report on outbreaks of contagious ovine digital dermatitis in Sweden. Acta Vet. Scan..

[25] Wilson-Welder J.H., Mansfield K., Han S., Bayles D.O., Alt D.P., Olsen S.C. (2022). Lesion Material From Treponema-Associated Hoof Disease of Wild Elk Induces Disease Pathology in the Sheep Digital Dermatitis Model. Front. Veter. Sci..

[26] Rasool A., Farooq S., Kumar S., Kashoo Z.A., Dar P.A., Bhat M.A., Qureshi S., Hussain I., Shah R.A., Taku A. (2023). Evidence of novel Treponema phylotypes implicated in contagious ovine digital dermatitis and association of treponemes with major lameness causing foot pathogens. Microb. Pathog..

[27] McPherson A.S., Whittington R.J., Hall E., Cook E.J., Jones J.V., Ang Y.Q., McTavish E.L., Dhungyel O.P. (2021). A comparison of mul-tivalent and bivalent vaccination strategies for the control of virulent ovine footrot. Vaccine.

[28] Albuquerque C., Cavaco S., Caetano P., Branco S., Monteiro H., Ramos M., Chimenos A.U., Leão C., Botelho A. (2022). Ovine footrot in Southern Portugal: Detection of *Dichelobacter nodosus* and *Fusobacterium necrophorum* in sheep with different lesion scores. Veter. Microbiol..

[29] Budnik M., Struck A.-K., Storms J., Wirth A., Jores J., Kuhnert P., Distl O. (2022). Serological Diversity of *Dichelobacter nodosus* in German Sheep Flocks. Animals.

[30] Lacombe-Antoneli A., Píriz S., Vadillo S. (2006). Aetiology of caprine footrot in Extremadura region, Spain. Acta Veter. Hung..

[31] Prosser N.S., Monaghan E.M., Green L.E., Purdy K.J. (2020). Serogroups of *Dichelobacter nodosus*, the cause of footrot in sheep, are randomly distributed across England. Sci. Rep..

[32] Wani S.A., Farooq S., Kashoo Z.A., Hussain I., Bhat M.A., Rather M.A., Aalamgeer S. (2019). Determination of prevalence, serological diversity, and virulence of *Dichelobacter nodosus* in ovine footrot with identification of its predominant serogroup as a potential vaccine candidate in J&K, India. Trop Anim Health Prod..

[33] Dhungyel O., Whittington R., Egerton J. (2002). Serogroup specific single and multiplex PCR with pre-enrichment culture and immuno-magnetic bead capture for identifying strains of *D. nodosus* in sheep with footrot prior to vaccination. Mol. Cell. Probes.

[34] McPherson A.S., Dhungyel O.P., Whittington R.J. (2018). Detection and Serogrouping of *Dichelobacter nodosus* Infection by Use of Direct PCR from Lesion Swabs To Support Outbreak-Specific Vaccination for Virulent Footrot in Sheep. J. Clin. Microbiol..

[35] Dhungyel O., Hunter J., Whittington R. (2014). Footrot vaccines and vaccination. Vaccine.

[36] Groenevelt M., Dekker C., Dhungyel O., Everts R., Hoogeveen J., Timmerman A., Zweerus H., Mokbel M., Duim B. (2023). Serogroups of *Dichelobacter nodosus* detected in footrot lesions in sheep using a new multiplex real-time PCR. Adv. Anim. Biosci..

[37] Dhungyel O., Schiller N., Eppleston J., Lehmann D., Nilon P., Ewers A., Whittington R. (2013). Outbreak-specific monovalent/bivalent vaccination to control and eradicate virulent ovine footrot. Vaccine.

[38] Gelasakis A.I., Kalogianni A.I., Bossis I. (2019). Aetiology, Risk Factors, Diagnosis and Control of Foot-Related Lameness in Dairy Sheep. Animals.

[39] Collee J.G., Watt B., Brown R., Johnstone S. (1974). The recovery of anaerobic bacteria from swabs. J. Hyg..

